# Effects of Dynamic Apnea Conditions on Performance, Kinematic Parameters, and Physiological Markers in 50 m Monofin Sprinting

**DOI:** 10.3390/sports14090406

**Published:** 2026-09-15

**Authors:** Gregory Kalaitzoglidis, Charoula Tsalouchou, George Tsalis

**Affiliations:** School of Physical Education and Sport Science at Serres, Aristotle University of Thessaloniki, 54124 Thessaloniki, Greece; gkalaitzoglidis@gmail.com (G.K.); xaratsalouxoy14@gmail.com (C.T.)

**Keywords:** finswimming, mammalian diving reflex, blood lactate, blood glucose, heart rate

## Abstract

Finswimming is a high-intensity aquatic sport in which performance is influenced by the interaction between biomechanical and physiological factors, particularly during underwater apnea sprint events. This study compared performance, kinematic characteristics, cardiovascular responses, metabolic responses, and perceived exertion during a maximal 50 m monofin sprint performed under three conditions: underwater apnea swimming (UW), surface swimming without a snorkel (SN), and surface swimming with a closed snorkel (SC). Ten highly trained young male national-level finswimmers completed a randomized repeated-measures protocol. Performance time, dolphin kick number and frequency, heart rate (HR), blood lactate concentration, blood glucose concentration, and rating of perceived exertion (RPE) were assessed. Sprint performance was significantly faster during UW (17.38 ± 1.32 s) than during both SN (19.20 ± 1.64 s) and SC (19.39 ± 1.53 s; *p* < 0.001), with a very large condition effect (ηp^2^ = 0.882). This was accompanied by fewer dolphin kicks during UW (40 ± 5) than during SN (44 ± 6) and SC (44 ± 5), with a large condition effect (ηp^2^ = 0.651), whereas kick frequency did not differ significantly among conditions (~137–140 kicks·min^−1^; ηp^2^ = 0.038). HR exhibited a biphasic recovery pattern across all conditions, declining rapidly from peak exercise values (HRpeak: ~169–171 bpm) to HRlow (~141–147 bpm) before increasing to HRhigh (~160–162 bpm) during early recovery, with a very large main effect of time (*p* < 0.001; ηp^2^ = 0.823). Post-exercise blood lactate concentration increased significantly in all conditions from ~1.3–1.5 to ~10.7–11.9 mmol·L^−1^ (*p* < 0.001), with a very large time effect (ηp^2^ = 0.954) and no significant between-condition differences. Blood glucose concentration (~5.4–5.9 mmol·L^−1^) and RPE (7–8 a.u.) showed no statistically significant condition-specific differences. In conclusion, underwater apnea swimming was associated with faster 50 m sprint performance and fewer dolphin kicks than the surface swimming conditions in highly trained young male national-level finswimmers. These performance and kick characteristics are consistent with potential biomechanical and hydrodynamic explanations described in previous research, although propulsive efficiency and hydrodynamic resistance were not directly measured in the present study. A biphasic HR recovery pattern was observed across conditions; however, the autonomic mechanisms underlying this response cannot be determined from the present measurements.

## 1. Introduction

Finswimming (FS) is a competitive aquatic sport characterized by using specialized equipment and distinct biomechanical demands. Monofin swimming (MF), one of the main disciplines of FS, involves a characteristic undulatory dolphin kick motion [1]. This technique requires coordinated movement of the trunk and lower limbs while maintaining the upper body in a streamlined hydrodynamic position [2,3]. Among FS events, the 50 m apnea is one of the most demanding, as athletes are required to complete the entire distance underwater without breathing and at maximal intensity [2]. This type of effort is physiologically defined as dynamic apnea [4].

Significant biomechanical and physiological differences exist between underwater and surface MF swimming. Underwater locomotion provides a hydrodynamic advantage due to the absence of wave drag, resulting in reduced active drag and improved mechanical efficiency [5]. As a result, underwater swimming velocity is generally higher compared to surface swimming [5,6]. In contrast, surface swimming involves continuous interaction with the air–water interface, leading to the generation of wave resistance and increased hydrodynamic drag [7]. The increased drag, together with disturbances in the swimmer’s streamlined body alignment, reduces mechanical efficiency compared to underwater swimming [5]. The use of fins can also influence swimming economy and mechanical efficiency [8]. Michalica et al. [9] reported that respiratory muscle training in a world-record-holding finswimmer was associated with an increased underwater distance and kick number while underwater velocity was maintained.

Heart rate (HR) is commonly used to characterize cardiovascular responses during 50 m apnea MF swimming [6,10]. Breath-hold conditions during dynamic exercise activate protective physiological mechanisms, primarily the diving response, which is characterized by bradycardia and peripheral vasoconstriction aimed at preserving oxygen supply to vital organs such as the brain [11,12,13]. However, during the 50 m apnea MF event, a physiological conflict occurs, as high-intensity muscular activity increases HR [5,6], whereas apnea and immersion induce bradycardia [14]. Kostoulas et al. [6] reported a rapid post-exercise HR decline followed by a secondary increase during recovery after high-intensity underwater apnea finswimming, suggesting a complex cardiovascular response during the transition from exercise to recovery.

Blood lactate (BL) concentration is commonly used as a metabolic marker during high-intensity exercise [15]. Blood glucose (BG) concentration was also assessed in the present study to characterize the acute metabolic response. At the cellular level, hypoxia activates hypoxia-inducible factor-1α (HIF-1α), which regulates glycolytic energy provision and promotes the expression of genes associated with glycolysis [16,17]. In addition, hypercapnia, characterized by elevated carbon dioxide (CO_2_) levels in the blood, may further contribute to these metabolic responses [17]. Burtscher et al. [16] suggested that increased blood lactate concentration under hypoxic conditions is related to activation of the sympathetic–adrenal system and enhanced glycogenolysis and glycolysis.

Despite the growing interest in apnea MF, limited evidence exists regarding performance and physiological responses under different apnea conditions. Underwater and surface apnea swimming have not been sufficiently compared in terms of heart rate responses, metabolic responses, and dolphin kick characteristics during maximal-intensity 50 m MF. Therefore, the aim of the present study was to compare performance and physiological responses during 50 m maximal-intensity apnea MF under three conditions: (a) underwater swimming, (b) surface swimming without a snorkel, and (c) surface swimming using a closed snorkel. The examined variables included swimming performance, dolphin kick number and frequency, heart rate, blood lactate and blood glucose concentrations, and rating of perceived exertion (RPE). It was hypothesized that underwater apnea swimming would result in superior performance and distinct physiological responses compared with the two surface apnea conditions.

## 2. Materials and Methods

### 2.1. Participants

Ten highly trained young male national-level finswimmers (n = 10; age: 16.3 ± 0.8 yrs; height: 1.75 ± 0.04 m; body mass: 73.5 ± 8.5 kg; competitive experience: 5.5 ± 0.5 yrs) participated in this study. The primary inclusion criterion was the ability to successfully complete a high-intensity 50 m apnea MF sprint. Participants were excluded if they reported a recent respiratory illness or had engaged in strenuous training within the 48 h preceding the first experimental session. All participants and their legal guardians were fully informed about the study purpose, procedures, risks, and benefits and provided written informed consent. The study was conducted in accordance with the Declaration of Helsinki and approved by the Local Ethics Research Committee of the School of Physical Education and Sport Science at Serres, Aristotle University of Thessaloniki (ERC-008/2025, 24 March 2025).

### 2.2. Experimental Design

A randomized repeated-measures design was used. The order of the three experimental conditions was determined for each participant by drawing lots. Each participant completed a single maximal-intensity 50 m MF sprint on three consecutive days under three apnea conditions: (a) underwater swimming (UW), (b) surface swimming without a snorkel (SN), and (c) surface swimming using a closed snorkel (SC). The SC condition was included to examine whether the presence of a snorkel and its associated additional air column and potential external resistance influence physiological and performance responses during surface swimming. In the SC condition, all participants swam at the surface while wearing the same front-mounted competition snorkel. The snorkel was sealed at its distal opening with adhesive and insulating tape to prevent airflow. It had an internal diameter of 23 mm and a length of 48 cm, resulting in an internal volume of approximately 199 mL, corresponding to the additional apparatus dead space.

### 2.3. Experimental Protocol

**Figure 1:**
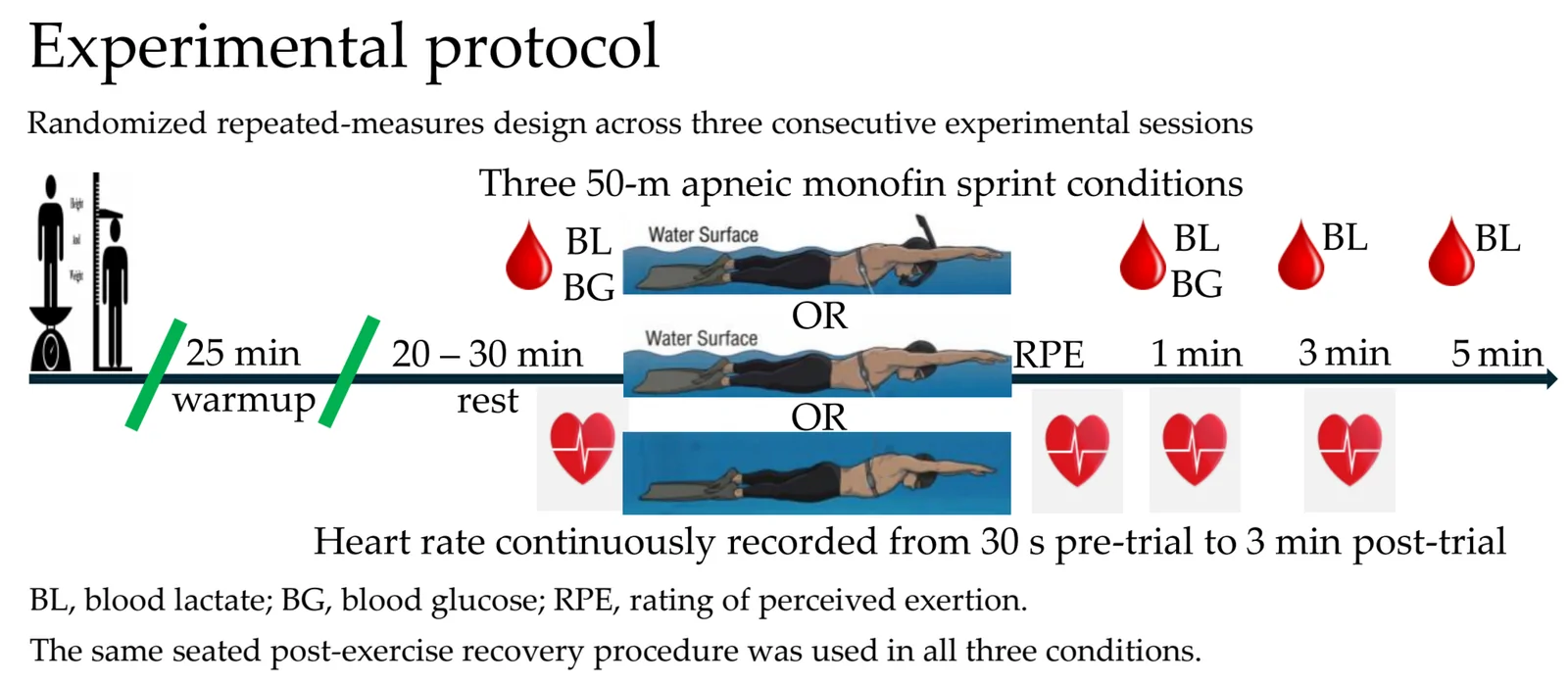
A schematic diagram of the experimental protocol.

All testing sessions were conducted in a 50 m outdoor swimming pool (water temperature: ~26 °C; ambient temperature: ~17 °C). Each session followed an identical standardized timeline. Upon arrival, participants completed a standardized 25 min warm-up. Within the warm-up protocol, 2 min passive recovery intervals were included between specific exercise components, as described below. The warm-up consisted of 1 × 200 m front crawl, followed by 2 min of passive recovery; 1 × 200 m swimming with bifins, followed by 2 min of passive recovery; 1 × 200 m monofin swimming, followed by 2 min of passive recovery; 4 × 15 m monofin finish sprints performed every 45 s, followed by 2 min of passive recovery; and 2 × 25 m maximal-intensity monofin swims performed without a start, commencing every 2 min. After completion of the entire warm-up protocol, participants underwent a separate 20–30 min passive recovery period before the experimental trial. Baseline physiological measurements (heart rate and capillary blood samples) were collected at the end of this 20–30 min recovery period and before the 50 m sprint. Anthropometric measurements (height and body mass) were performed during the first visit prior to the experimental trials using a wall-mounted stadiometer (Seca 206, Hamburg, Germany) and a digital scale (Seca 813, Hamburg, Germany), respectively. Each 50 m sprint was initiated with an in-water push-off and terminated upon wall contact at the 50 m mark. Immediately after completing the 50 m sprint, participants exited the pool and sat on the pool deck, where they remained seated throughout the post-exercise recovery period. The same recovery procedure was followed in all three experimental conditions. Participants were instructed to perform maximal effort under voluntary apnea conditions in all trials. A schematic diagram of the experimental protocol is presented in Figure 1.

### 2.4. Performance and Kinematic Measures

Swimming performance (SP) was defined as the time required to complete the 50 m sprint. Sprint time was recorded using three digital hand-held stopwatches (SL 929-R, Oregon Scientific, Tualatin, OR, USA) operated by three experienced certified timekeepers.

The final performance time was calculated as the mean of the three independently recorded values. Inter-timekeeper variability was consistently below 3%, indicating high agreement among the three timekeepers. Dolphin kick number (DKN) was independently assessed by the same three experienced certified timekeepers during each trial, with the total number of dolphin kicks recorded for each participant. The mean of the three recorded DKN values was used for subsequent analysis. DKN assessment was based on direct observation; no video-based or blinded video assessment was performed, and no formal inter-rater reliability statistic was calculated. Subsequently, dolphin kick frequency (DKF; kicks·min^−1^) was calculated. Swimming depth during the underwater condition was recorded at the 25 m mark using a submerged action camera (GoPro Hero 9, San Mateo, CA, USA), providing a representative depth value during the trial [18].

### 2.5. Physiological Measurements

HR was continuously recorded using a telemetry chest strap (Polar H10, Polar Electro Oy, Kempele, Finland) from 30 s pre-exercise until 3 min post-exercise. HR values were recorded at 1 s intervals throughout the monitoring period. For graphical presentation, mean HR values were plotted at 30 s before swimming, immediately before the start of swimming, at HRpeak, HRlow, HRhigh, and at 1, 2, and 3 min after swimming. HRpeak was defined as the maximum HR attained during the swimming trial, whereas HRlow and HRhigh were participant-specific values representing the minimum HR during early recovery (1–25 s post-test) and the subsequent higher HR value (2–40 s post-test), respectively. Because HRlow and HRhigh occurred at participant-specific times, these points represent physiological response characteristics rather than fixed post-exercise sampling times. Successive mean HR values were connected by lines solely to facilitate visualization of the temporal response pattern; no trend-line fitting, regression analysis, or mathematical curve-fitting procedure was applied. Post-exercise HR monitoring was therefore performed while participants remained seated on the pool deck, as described in the experimental protocol. HR data were analyzed continuously and expressed as mean and peak values.

Capillary blood samples were obtained from the fingertip to determine BL (Lactate Scout 4, EKF Diagnostics, Leipzig, Germany; measurement range: 0.5–25.0 mmol·L^−1^; sample volume: 0.2 μL) and BG concentrations (Multicare-in, Biochemical Systems International, Arezzo, Italy; measurement range: 0.6–33.3 mmol·L^−1^; sample volume: 0.5 μL). Baseline samples were collected prior to each sprint. Post-exercise blood lactate concentration was measured at the 1st, 3rd, and 5th minutes of recovery to determine peak values, whereas blood glucose concentration was assessed at the 1st minute of recovery.

### 2.6. Perceptual Measures

RPE was assessed immediately after each sprint using the Borg 10-point scale, providing an integrated assessment of exercise intensity [19].

### 2.7. Standardization Procedures

Participants were instructed to maintain their normal diet throughout the experimental period. Following the initial 48 h restriction on strenuous training applied before the first experimental session as an eligibility criterion, participants were instructed to avoid strenuous physical activity for at least 12 h before each of the two subsequent experimental sessions. All trials were performed under identical environmental and pool conditions to minimize external variability.

### 2.8. Statistical Analysis

An a priori power analysis was performed using G*Power (v. 3.1.9.7, Universität Düsseldorf, Germany) for a repeated-measures ANOVA (three conditions, effect size partial η^2^ = 0.13, α = 0.05, power = 0.80), indicating a minimum required sample size of nine participants [20]. Accordingly, ten participants were recruited to ensure adequate statistical power. Data normality was assessed using the Shapiro–Wilk test. Sphericity was evaluated using Mauchly’s test for within-subject effects with more than two levels. No significant violations of sphericity were detected (all *p* > 0.05). For within-subject factors with only two levels, sphericity is inherently satisfied. No Greenhouse–Geisser correction was required for the reported analyses. Because all experimental conditions were assessed within the same participants and no independent groups were compared, a between-group homogeneity-of-variance test was not applicable. A one-way repeated-measures ANOVA was used to compare SP, DKN, DKF, and RPE across the three conditions. A two-way repeated-measures ANOVA (condition × time) was applied for HR, BL, and BG concentrations. When a significant main effect or interaction was observed, Bonferroni-adjusted post hoc tests were performed. Statistical significance was set at *p* < 0.05. Data are presented as mean ± standard deviation (SD) for descriptive purposes and, where appropriate, as estimated means with 95% confidence intervals (CIs). Effect sizes are reported as partial eta squared (ηp^2^), together with two-sided 95% confidence intervals estimated using the noncentral F distribution. All analyses were performed using SPSS version 27.0 (IBM Corp., Armonk, NY, USA).

## 3. Results

All ten participants completed all three experimental conditions, and no dropouts occurred.

### 3.1. Performance and Kinematic Parameters

**Figure 2:**
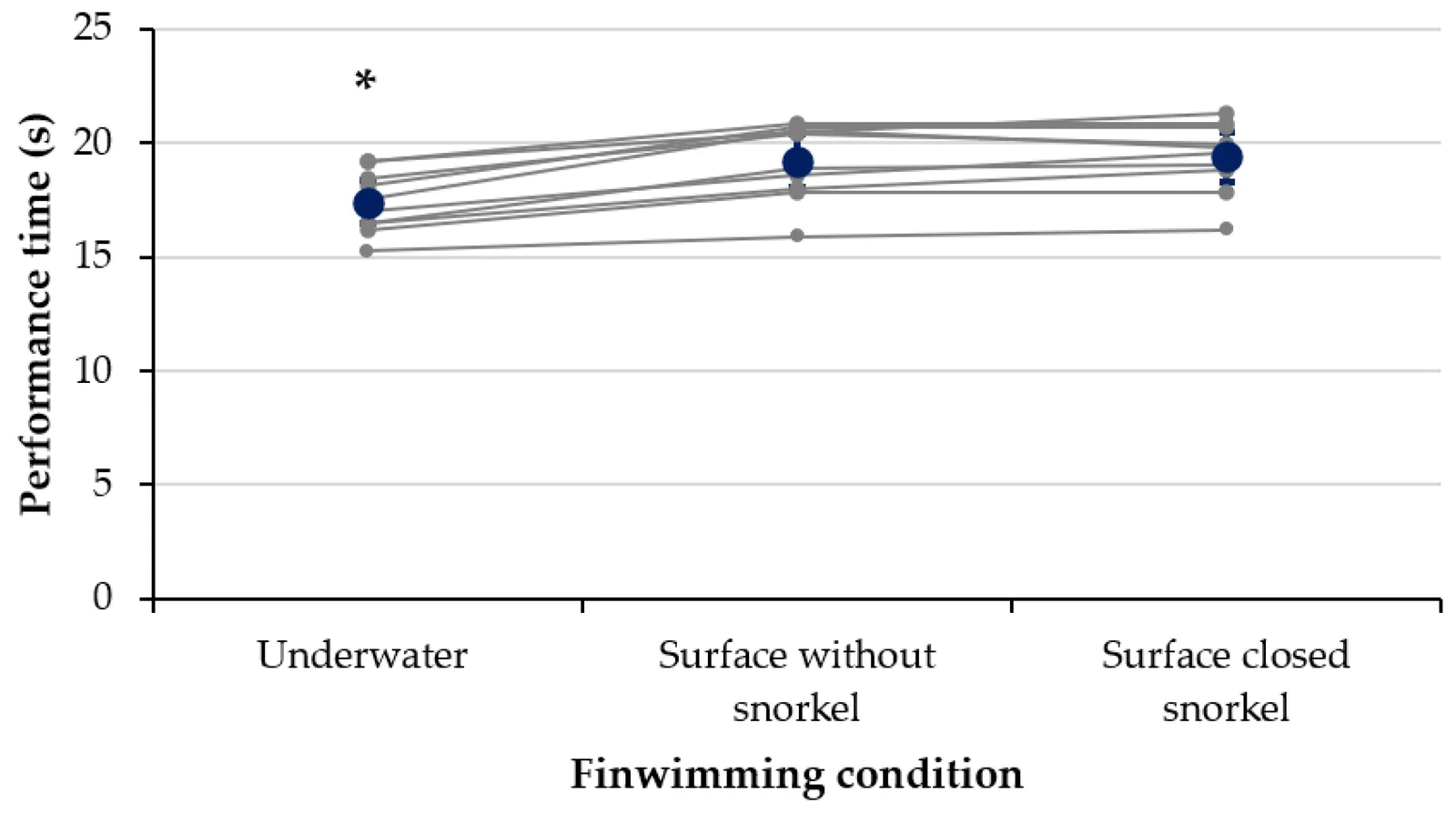
Individual performance times across the underwater swimming (UW), surface swimming without a snorkel (SN), and surface swimming using a closed snorkel (SC) conditions. Gray lines and markers represent individual participant trajectories, while blue markers and error bars represent condition means and 95% confidence intervals, respectively. * Indicates a statistically significant difference between UW and both SN and SC (*p* < 0.001).

A significant main effect of swimming condition was observed for performance time (F_2,18_ = 67.31, *p* < 0.001, ηp^2^ = 0.882, 95% CI [0.746, 0.933]). The UW condition resulted in significantly faster performance compared with both surface conditions (SN and SC) (17.38 s, 95% CI [16.44, 18.33] vs. 19.20 s, 95% CI [18.03, 20.38] and 19.39 s, 95% CI [18.29, 20.48], respectively; *p* < 0.001) (Figure 2).

**Figure 3:**
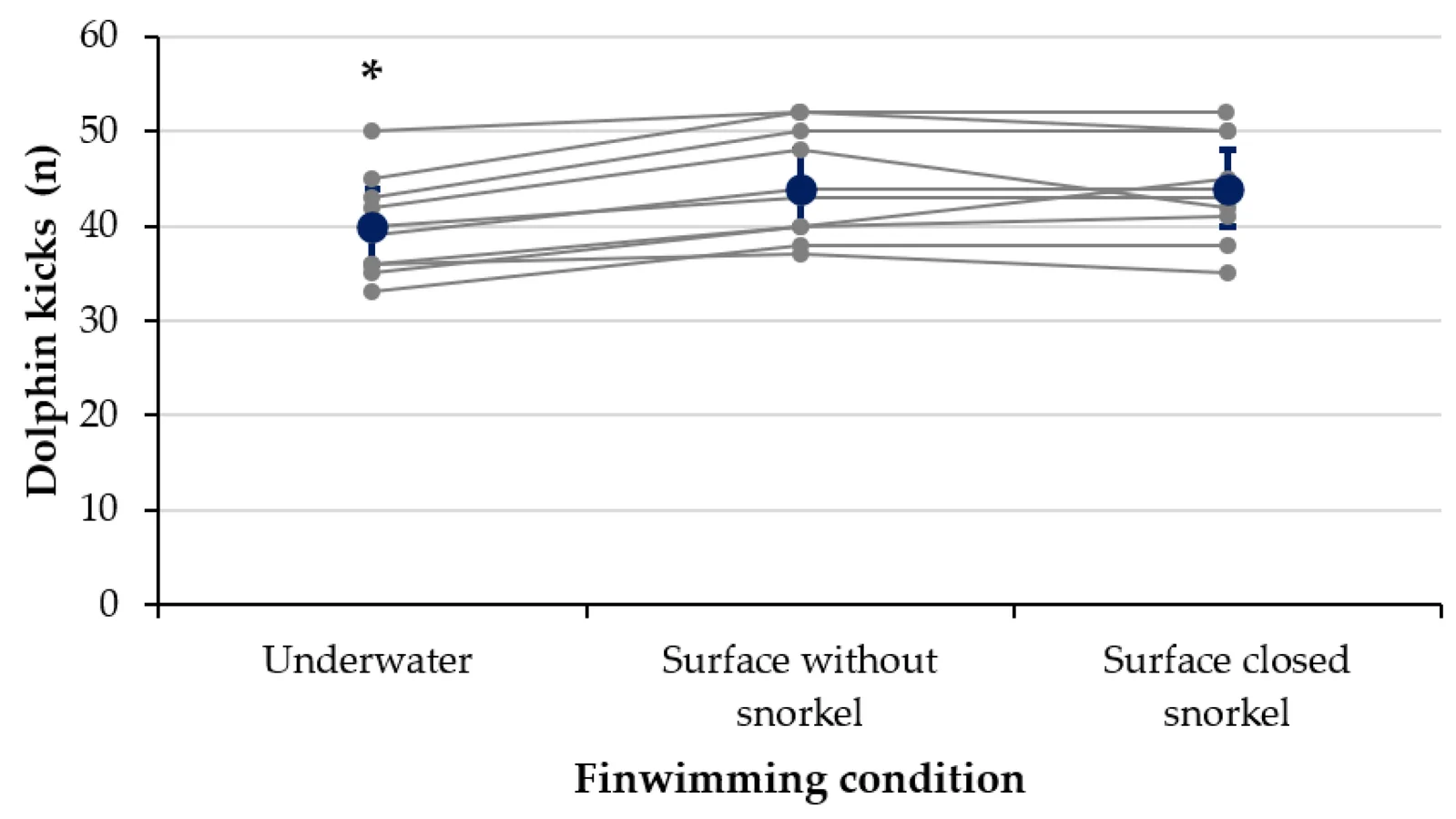
Individual dolphin kick numbers (DKN) across the underwater swimming (UW), surface swimming without a snorkel (SN), and surface swimming using a closed snorkel (SC) conditions. Gray lines and markers represent individual participant trajectories, while blue markers and error bars represent condition means and 95% confidence intervals, respectively. * Indicates statistically significant differences between UW and SN (*p* < 0.001) and between UW and SC (*p* = 0.009).

Similarly, the DKN was significantly affected by swimming condition (F2,18 = 16.77, p < 0.001, ηp2 = 0.651, 95% CI [0.318, 0.797]). Participants performed significantly fewer kicks during the UW condition (40 kicks, 95% CI [36, 43]) compared with both the SN (44 kicks, 95% CI [40, 48], p < 0.001) and SC (44 kicks, 95% CI [40, 48], p = 0.009) conditions (Figure 3).

**Figure 4:**
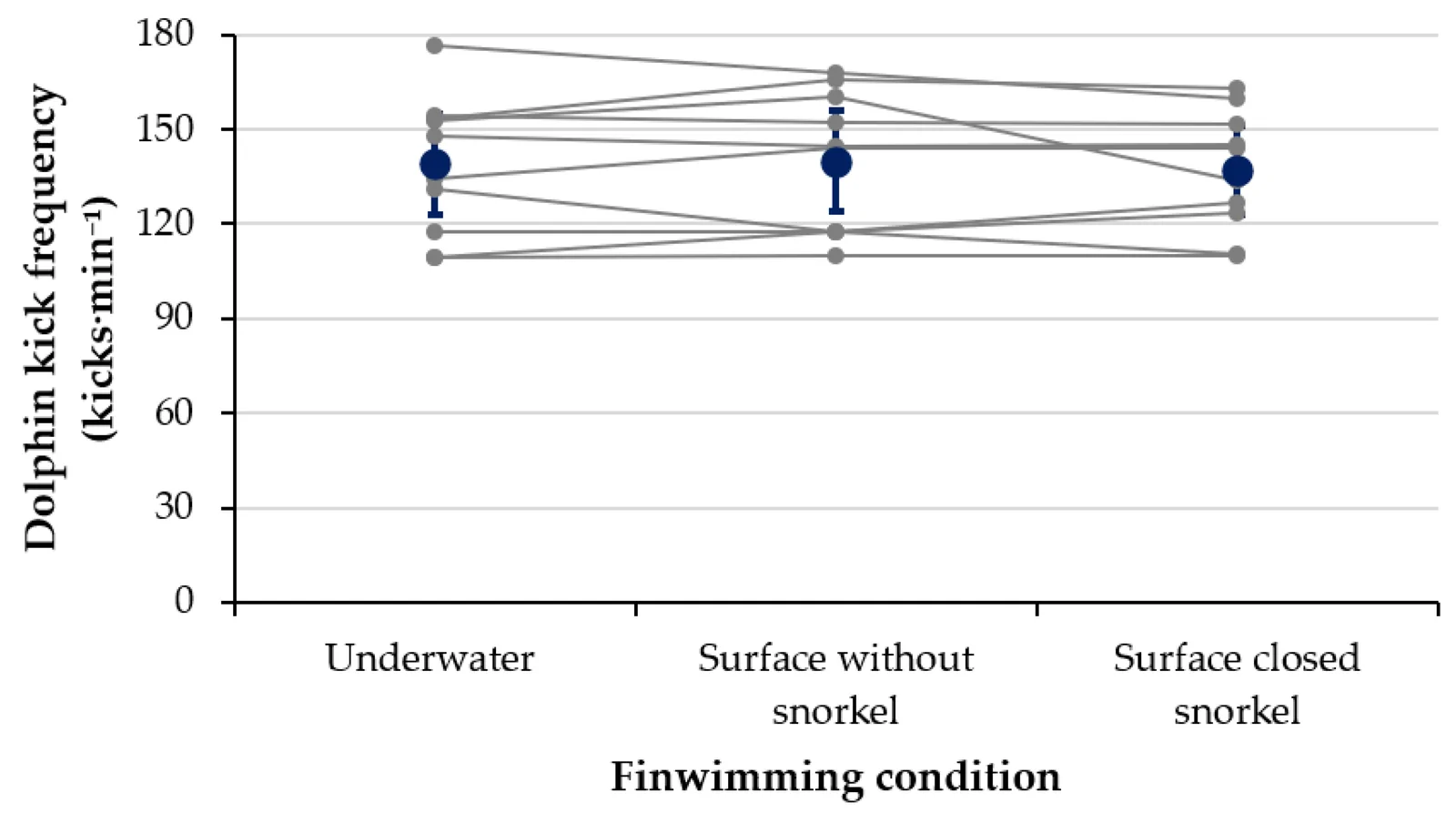
Individual dolphin kick frequencies (DKF) across the underwater swimming (UW), surface swimming without a snorkel (SN), and surface swimming using a closed snorkel (SC) conditions. Gray lines and markers represent individual participant trajectories, while blue markers and error bars represent condition means and 95% confidence intervals, respectively.

In contrast, DKF did not differ significantly among conditions (F_2_,_18_ = 0.36, *p* = 0.705, ηp^2^ = 0.038, 95% CI [0.000, 0.248]), with comparable values observed for UW (139 kicks·min−1, 95% CI [123, 154]), SN (140 kicks·min−1, 95% CI [124, 156]), and SC (137 kicks·min^−1^, 95% CI [123,151]) (Figure 4).

### 3.2. Physiological Markers

#### 3.2.1. Heart Rate

**Figure 5:**
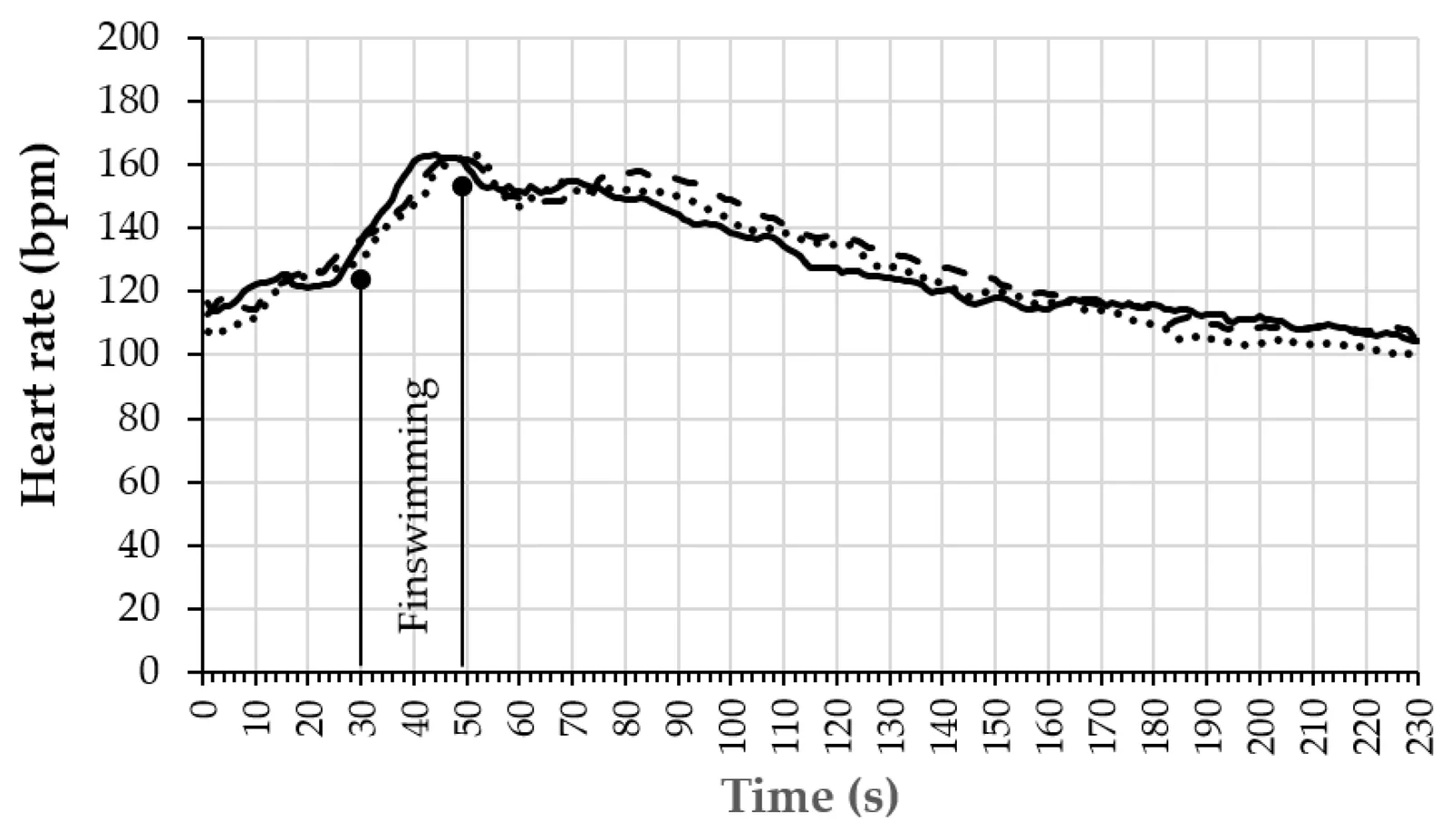
Heart rate responses under the three swimming conditions: underwater swimming (UW—solid black line), surface swimming without a snorkel (SN—dashed black line), and surface swimming using a closed snorkel (SC—dotted black line). HR values were recorded at 1 s intervals, and each plotted value represents the group mean HR at the corresponding second for each condition. The *x*-axis represents time in seconds, with grid lines displayed at regular intervals to facilitate interpretation. The two vertical black lines indicate the mean finswimming time.

Figure 5 presents the mean values of HR changes 30 s before exercise, immediately before the sprint, during the exercise phase (approximately from 30 to 50 s), and during the 3 min post-exercise recovery period across the three experimental conditions.

**Figure 6:**
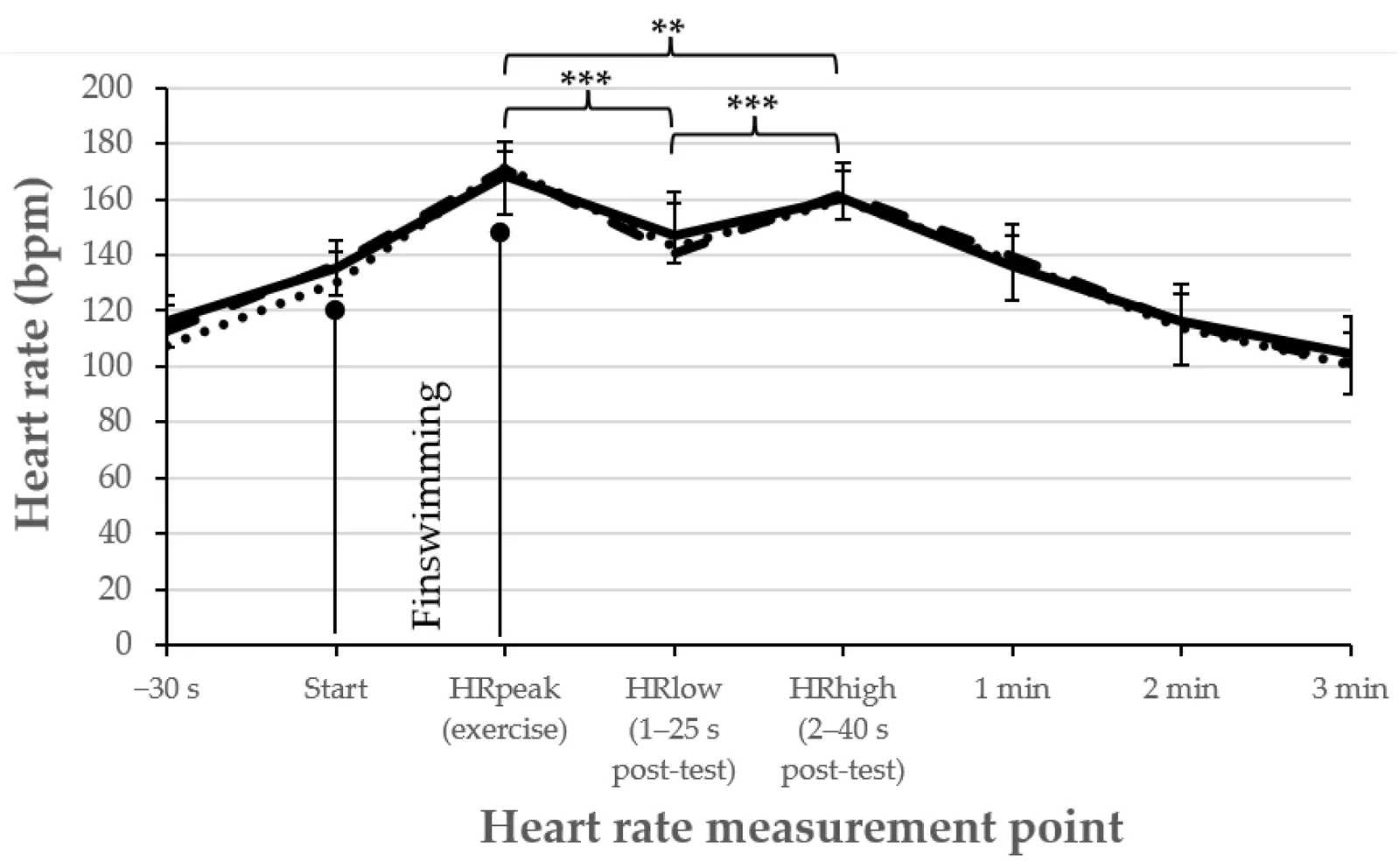
Heart rate responses across the three swimming conditions: underwater swimming (UW—solid black line), surface swimming without a snorkel (SN—dashed black line), and surface swimming using a closed snorkel (SC—dotted black line). The *x*-axis represents HR measurement points: 30 s before swimming, the start of swimming, HRpeak, HRlow, HRhigh, and 1, 2, and 3 min after swimming. HRpeak represents the maximum HR attained toward the end of the swimming trial, whereas HRlow and HRhigh represent participant-specific minimum and subsequent higher HR values during early recovery, occurring 1–25 s and 2–40 s post-test, respectively. The two black vertical lines indicate the mean swimming time. Lines connecting successive mean HR values are included for visualization of the response pattern only and do not represent fitted trend lines or regression models. Error bars represent the standard deviation. Horizontal brackets indicate statistically significant pairwise differences among HRpeak, HRlow, and HRhigh following Bonferroni adjustment (*** *p* ≤ 0.001; ** *p* = 0.016).

Based on the findings of Kostoulas et al. [6], showing a secondary increase in HR with a second higher level occurring within the first 30 s of recovery after the initial rapid post-exercise decline in apnea swimming, HRlow and HRhigh values were extracted during the first minute of recovery for each swimmer. The results are presented in Figure 6.

For statistical analysis, HRpeak obtained during exercise, HRlow recorded during early recovery (1–25 s post-test), and HRhigh recorded during the subsequent early-recovery increase (2–40 s post-test) were analyzed. The mean difference between HRlow and HRhigh was 17 bpm, with a mean temporal delay of 10 s.

No significant condition × time interaction was observed (F_4,36_ = 0.92, *p* = 0.465, ηp^2^ = 0.092, 95% CI [0.000, 0.234]). A significant main effect of time was found (F_2,18_ = 41.85, *p* < 0.001, ηp^2^ = 0.823, 95% CI [0.625, 0.898]), whereas no differences between conditions were detected (F_2,18_ = 0.03, *p* = 0.972, ηp^2^ = 0.003, 95% CI [0.000, 0.012]).

Overall, HR differed significantly across HRpeak, HRlow, and HRhigh within all conditions: UW, 169 bpm, 95% CI [159, 178] → 147 bpm, 95% CI [140, 154] → 160 bpm, 95% CI [155, 165]; SN, 171 bpm, 95% CI [166, 175] → 141 bpm, 95% CI [128, 153] → 162 bpm, 95% CI [154, 170]; and SC, 171 bpm, 95% CI [164, 178] → 143 bpm, 95% CI [129, 157] → 160 bpm, 95% CI [152, 167] (p < 0.001). Bonferroni-adjusted pairwise comparisons showed significant differences between HRpeak and HRlow (p < 0.001), HRpeak and HRhigh (p = 0.016), and HRlow and HRhigh (*p* = 0.001) (Figure 6).

#### 3.2.2. Blood Lactate and Blood Glucose

**Figure 7:**
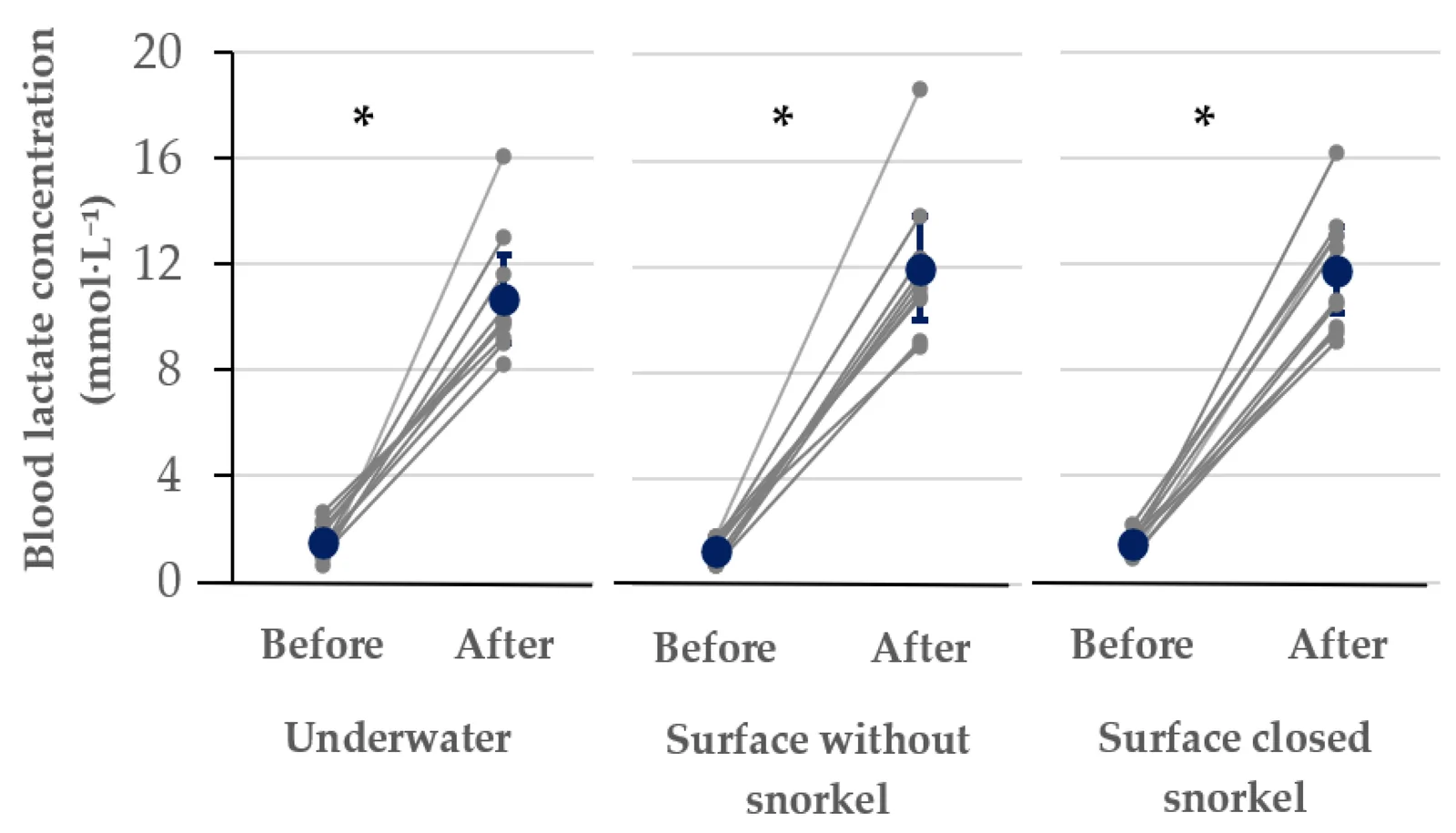
Individual blood lactate concentrations before and after swimming across the UW, SN, and SC conditions. Gray lines and markers represent individual participant trajectories, while blue markers and error bars represent condition means and 95% confidence intervals, respectively. * Indicates a statistically significant difference in blood lactate concentrations between before and after the trials (*p* < 0.001).

The results showed no significant condition × time interaction for BL concentrations, F_2,18_ = 3.42, *p* = 0.055, ηp^2^ = 0.276, 95% CI [0.000, 0.539], whereas a significant main effect of time was observed, F_1,9_ = 186.62, *p* < 0.001, ηp^2^ = 0.954, 95% CI [0.850, 0.978]. Post-swimming blood lactate concentrations were significantly higher compared with pre-trial values in all conditions [UW (1.52 mmol·L^−1^, 95% CI [1.06, 1.98]) vs. 10.68 mmol·L^−1^, 95% CI [9.01, 12.35]), SN (1.29 mmol·L^−1^, 95% CI [0.95, 1.63]) vs. 11.93 mmol·L^−1^, 95% CI [9.95, 13.91]), and SC (1.44 mmol·L^−1^, 95% CI [1.15, 1.73]) vs. 11.76 mmol·L^−1^, 95% CI [10.13, 13.39]), with no significant differences between swimming conditions, F_2,18_ = 1.37, *p* = 0.279, ηp^2^ = 0.132, 95% CI [0.000, 0.400] (Figure 7).

**Figure 8:**
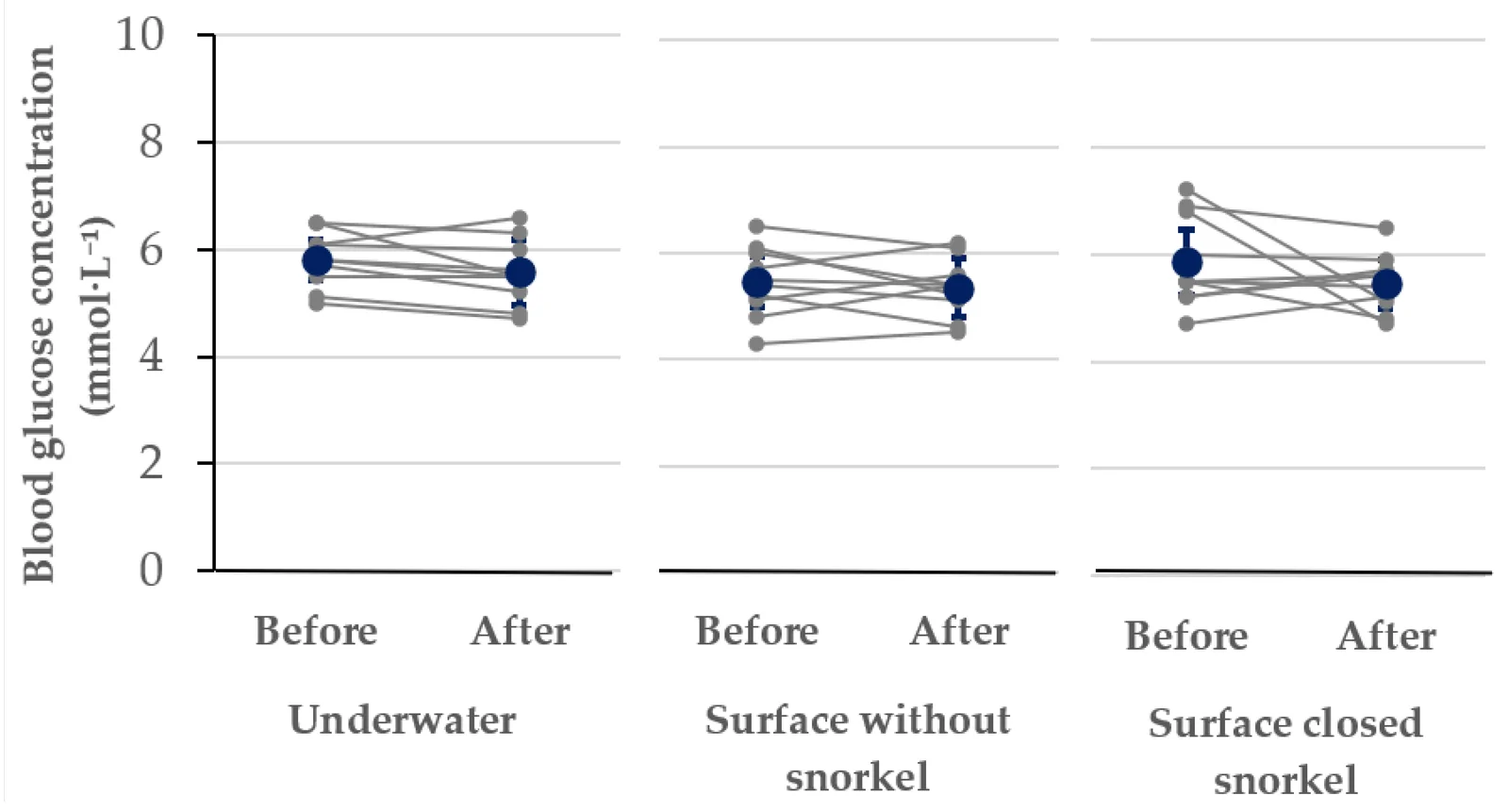
Individual blood glucose concentrations before and after swimming across the UW, SN, and SC conditions. Gray lines and markers represent individual participant trajectories, while blue markers and error bars represent condition means and 95% confidence intervals, respectively.

For BG, no statistically significant condition × time interaction was detected (F_2,18_ = 0.46, *p* = 0.637, ηp^2^ = 0.049, 95% CI [0.000, 0.274]), nor was there a significant main effect of time (F_1,9_ = 4.02, *p* = 0.076, ηp^2^ = 0.309, 95% CI [0.000, 0.654]). BG concentrations before and after swimming were UW: 5.81 mmol·L^−1^, 95% CI [5.44, 6.18] vs. 5.57 mmol·L^−1^, 95% CI [5.14, 6.00]; SN: 5.46 mmol·L^−1^, 95% CI [4.99, 5.93] vs. 5.35 mmol·L^−1^, 95% CI [4.96, 5.74]; and SC: 5.85 mmol·L^−1^, 95% CI [5.25, 6.45] vs. 5.45 mmol·L^−1^, 95% CI [5.07, 5.83]. No statistically significant main effect of swimming condition was detected (F_2,18_ = 1.32, *p* = 0.292, ηp^2^ = 0.128, 95% CI [0.000, 0.394]) (Figure 8).

### 3.3. Rating of Perceived Exertion

**Figure 9:**
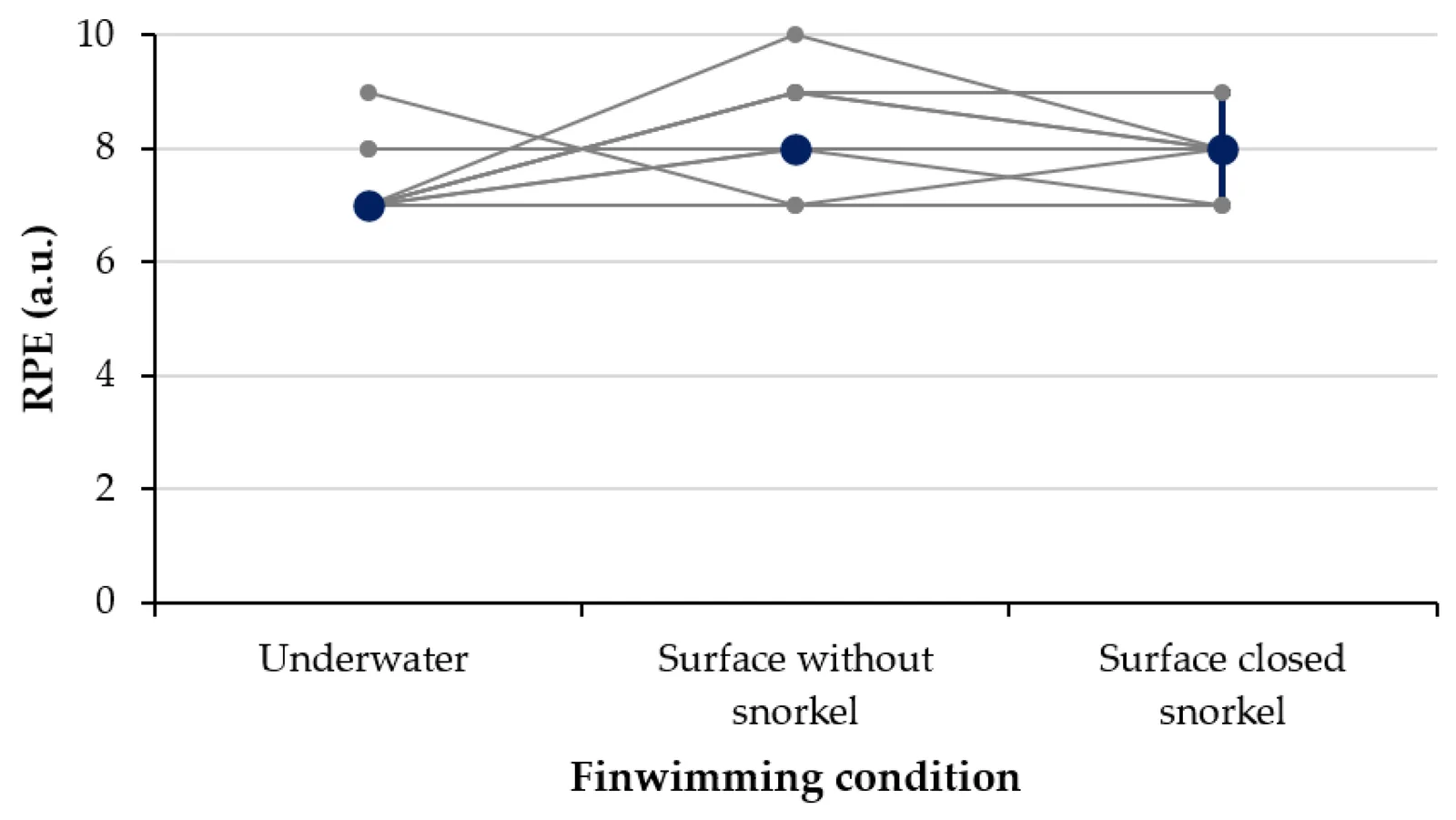
Individual ratings of perceived exertion (RPE) across the UW, SN, and SC conditions. Gray lines and markers represent individual participant trajectories, while blue markers and error bars represent condition means and 95% confidence intervals, respectively.

Finally, no statistically significant effect of swimming condition was observed for RPE (F_2,18_ = 3.25, *p* = 0.062, ηp^2^ = 0.265, 95% CI [0.000, 0.530]). Perceived exertion remained at similar levels across the three swimming conditions: UW (7 a.u., 95% CI [7, 8]), SN (8 a.u., 95% CI [8, 9]), and SC (8 a.u., 95% CI [7, 8]) (Figure 9).

## 4. Discussion

The present study examined the effects of underwater apnea swimming compared with two surface swimming conditions on performance, kinematic characteristics, heart rate responses, blood lactate and blood glucose concentrations, and perceived exertion during a maximal 50 m effort. The main findings of the study were: (a) the significantly faster performance achieved during UW swimming, accompanied by a lower number of kicks without changes in kick frequency, and (b) the distinct biphasic heart rate recovery response observed after exercise in all conditions, characterized by an initial rapid post-exercise decline followed by a secondary heart rate increase within the first minute of recovery.

### 4.1. Performance and Kinematic Parameters

The faster performance observed during UW, together with the lower number of dolphin kicks and comparable kick frequency, represents the main performance-related finding of the present study. The magnitude of the condition effect on performance time was substantial (ηp^2^ = 0.882, 95% CI [0.746, 0.933]), as was the effect on dolphin kick number (ηp^2^ = 0.651, 95% CI [0.318, 0.797]). In contrast, the effect of condition on dolphin kick frequency was small and non-significant (ηp^2^ = 0.038, 95% CI [0.000, 0.248]). Thus, the faster UW performance was accompanied by fewer propulsive cycles without a corresponding increase in kicking rate. However, this finding alone does not demonstrate greater propulsion per kick or improved propulsive efficiency.

The observed performance and kick characteristics are consistent with potential biomechanical and hydrodynamic explanations described in previous studies. Fully submerged swimming has been associated with reduced wave-making resistance compared with surface swimming [5,7,21,22,23,24,25], and the mean swimming depth of approximately 1.3 m (0.9–1.6 m) observed at mid-distance in the present study is compatible with a fully submerged swimming position. Previous studies of underwater undulatory swimming have also highlighted the potential importance of body alignment and reduced hydrodynamic resistance for sprint swimming performance [22,23,24]. In addition, previous observations in elite finswimming have shown that changes in underwater distance and kick characteristics can occur without compromising underwater velocity [9]. Nevertheless, wave-making resistance, active drag, body alignment, kick amplitude, kick length, instantaneous swimming velocity, and propulsive efficiency were not directly measured in the present study. Therefore, the present findings cannot establish reduced hydrodynamic resistance, greater propulsion per kick, or enhanced propulsive efficiency as mechanisms responsible for the faster UW performance. These factors should instead be regarded as plausible explanations that require direct biomechanical and hydrodynamic assessment.

No significant performance difference was observed between the two surface conditions (SN and SC), while DKN and DKF were also broadly comparable between these conditions. This finding indicates that, under the present experimental protocol, the presence of the sealed snorkel was not associated with a detectable change in sprint performance or the measured kick characteristics compared with surface swimming without a snorkel. However, because the additional hydrodynamic resistance imposed by the snorkel was not directly quantified, the absence of a statistically significant difference should not be interpreted as evidence that the device had no hydrodynamic effect. Overall, the faster performance observed during UW relative to both surface conditions is consistent with a potential contribution of submergence-related biomechanical and hydrodynamic factors to sprint performance, but the specific mechanisms responsible for this advantage cannot be determined from the measurements obtained in the present study.

### 4.2. Physiological Markers

#### 4.2.1. Heart Rate

A notable physiological finding of the present study was the biphasic HR recovery response observed during the early post-exercise period. Specifically, HR exhibited a rapid decline immediately after exercise cessation (HRlow), followed by a delayed secondary increase (HRhigh) approximately 10 s later, with a mean difference of 17 bpm between the two phases. A biphasic HR recovery pattern was observed across all three experimental conditions. The magnitude of the time effect was very large (ηp^2^ = 0.823, 95% CI [0.625, 0.898]), supporting a pronounced temporal change in HR across the recovery period. In contrast, the condition × time interaction showed a substantially smaller effect (ηp^2^ = 0.092, 95% CI [0.000, 0.234]), while the main effect of condition was negligible (ηp^2^ = 0.003, 95% CI [0.000, 0.012]). These findings indicate that the primary feature of the response was the temporal change in HR during recovery rather than a condition-specific difference in the magnitude of this response.

The observed biphasic HR recovery pattern represents a temporal response following maximal swimming exercise. The initial rapid decline in HR after exercise cessation is consistent with the expected reduction in cardiovascular demand during early post-exercise recovery. Previous studies incorporating measures of autonomic regulation have attributed early HR recovery partly to cardiac parasympathetic reactivation [26,27,28]. However, HRV or other direct indices of autonomic regulation were not assessed in the present study; therefore, autonomic regulation cannot be inferred directly from the observed HR decline. In apnea conditions, additional cardiovascular adjustments associated with the human diving response may also contribute [29,30], although their specific contribution cannot be determined from the present measurements.

The subsequent increase in HR observed during early recovery may similarly reflect the complex interaction between cardiovascular recovery and the physiological demands persisting after maximal breath-hold exercise. Previous research has suggested that metabolic and respiratory factors may contribute to post-exercise cardiovascular responses [27,28]; however, the present study did not include measurements of oxygen saturation, ventilation, blood gases, or other direct indicators of these processes. Therefore, the secondary HR increase should be interpreted as an observed temporal characteristic of recovery rather than as direct evidence of a change in sympathetic activity or a specific autonomic mechanism.

Importantly, the biphasic HR pattern was observed across all three experimental conditions, suggesting that this response may represent a general feature of recovery following maximal-intensity swimming rather than a condition-specific effect of underwater apnea. The present findings extend the observations of Kostoulas et al. [6], who previously reported a secondary HR increase during early recovery following apnea swimming, while highlighting the need for future studies combining continuous HR monitoring with HRV, oxygen saturation, respiratory, and blood-gas measurements to determine the physiological mechanisms underlying this response.

#### 4.2.2. Blood Lactate and Blood Glucose

Metabolically, all experimental conditions elicited a pronounced post-exercise increase in blood lactate concentration, consistent with substantial anaerobic glycolytic involvement during the 50 m sprint effort.

The magnitude of the main effect of time on blood lactate concentration was very large (ηp^2^ = 0.954, 95% CI [0.850, 0.978]), reflecting the pronounced post-exercise increase. The condition × time interaction did not reach statistical significance (*p* = 0.055), although the effect-size estimate was non-trivial and imprecise (ηp^2^ = 0.276, 95% CI [0.000, 0.539]). The main effect of swimming condition was also not statistically significant (ηp^2^ = 0.132, 95% CI [0.000, 0.400]). Although the point estimate was non-trivial, the wide confidence interval included zero, indicating substantial uncertainty regarding the magnitude of the condition effect. Given the relatively small sample size and the wide confidence intervals, condition-specific differences in blood lactate responses cannot be excluded. This response is consistent with the substantial anaerobic contribution expected during short-duration maximal exercise, in which anaerobic glycolysis contributes substantially to energy provision [31,32]. Importantly, despite the superior performance observed in underwater conditions, the increase in blood lactate concentration did not differ significantly between conditions, indicating that the faster UW performance was not accompanied by a greater blood lactate response.

The absence of statistically significant between-condition differences in blood lactate concentration may reflect the high metabolic demand imposed by maximal sprint swimming across all modalities. This interpretation is consistent with the substantial anaerobic contribution generally reported during short-duration maximal exercise [32,33]. Although UW resulted in faster performance, the present metabolic measurements do not identify the mechanism responsible for this performance difference. The lack of a statistically significant main effect of condition on blood lactate concentration indicates that no clear between-condition difference in blood lactate response was detected in the present sample. The superior UW performance may be compatible with biomechanical and hydrodynamic factors described in previous research; however, these mechanisms were not directly quantified and therefore cannot be established from the present data.

For blood glucose concentration, no statistically significant condition × time interaction (*p* = 0.637, ηp^2^ = 0.049, 95% CI [0.000, 0.274]), main effect of time (*p* = 0.076, ηp^2^ = 0.309, 95% CI [0.000, 0.654]), or main effect of condition (*p* = 0.292, ηp^2^ = 0.128, 95% CI [0.000, 0.394]) was detected. The condition × time effect estimate was relatively small, whereas the time and condition effect estimates were larger; however, all associated confidence intervals were wide and included zero, indicating considerable uncertainty regarding the magnitude of these effects. Therefore, the present findings should not be interpreted as definitive evidence of an absence of a blood glucose response. Therefore, the present findings should not be interpreted as definitive evidence of an absence of a blood glucose response. Rather, blood glucose concentrations appeared broadly comparable across measurement times and swimming conditions in the present sample. Given the extremely short duration of the exercise bout (17–20 s), these findings are expected, as such efforts predominantly rely on intramuscular phosphagen stores and anaerobic glycolysis, while hepatic glycogenolysis and systemic glucose mobilization may not be sufficient to produce measurable changes in blood glucose concentration [32,34].

### 4.3. Rating of Perceived Exertion

No statistically significant effect of swimming condition on RPE was detected (*p* = 0.062, ηp^2^ = 0.265, 95% CI [0.000, 0.530]). Although RPE values were broadly comparable across conditions, the effect-size estimate was non-trivial (ηp^2^ = 0.265), but its wide confidence interval (95% CI [0.000, 0.530]) indicates considerable uncertainty, particularly given the relatively small sample size. Therefore, these findings should not be interpreted as definitive evidence of an absence of condition-related differences in perceived exertion. RPE provides an integrated subjective assessment of exercise intensity and physiological strain within the psychophysical framework originally proposed by Borg [19], and the validity of the Borg RPE scale as an indicator of exercise intensity has been supported by subsequent meta-analytic evidence [35]. Importantly, the faster performance observed during UW was not accompanied by a statistically significant increase in RPE; however, the present data do not allow this finding to be attributed to improved mechanical or propulsive efficiency.

### 4.4. Limitations and Future Directions

Several limitations should be considered when interpreting the present findings. First, the relatively small and homogeneous sample of ten highly trained male national-level finswimmers limits the generalizability of the results to female athletes, athletes of different competitive levels, and other age or training groups. Although the a priori power analysis indicated that the sample size was adequate for the repeated-measures design, smaller effects may not have been detected, and the possibility of Type II error cannot be excluded.

Second, sprint performance was assessed using manual timing rather than an automated electronic system. Although three experienced certified timekeepers independently recorded each trial and the mean value was used, electronic timing would provide greater temporal precision. In addition, the biomechanical assessment was limited to dolphin kick number, kick frequency, and swimming depth at the 25 m point. Although dolphin kick number was independently assessed by three experienced certified timekeepers and the mean value was used for analysis, no formal inter-rater reliability analysis or blinded video-based assessment was performed. This should be considered when interpreting the dolphin kick results. Variables such as kick amplitude, kick length, instantaneous swimming velocity, body alignment, hydrodynamic resistance, and propulsive efficiency were not directly measured. Therefore, biomechanical and hydrodynamic mechanisms potentially contributing to the faster UW performance cannot be established from the present data and should be examined using continuous kinematic and hydrodynamic measurements in future studies.

Third, direct physiological markers of autonomic and respiratory regulation, including HRV, oxygen saturation, ventilation, and blood gases, were not assessed. Consequently, the mechanisms underlying the observed biphasic HR recovery response cannot be determined from the present measurements, and any mechanistic interpretation of this response remains speculative. In addition, participants exited the pool and assumed a seated position immediately after each sprint; although this procedure was standardized across conditions, this transition may have influenced very early post-exercise HR kinetics. The biphasic response should therefore be interpreted as an observed temporal HR pattern under the present experimental protocol rather than as evidence of a specific autonomic mechanism. Finally, the study examined only short-duration maximal 50 m efforts, and future research should determine whether these findings extend to other swimming distances, intensities, underwater durations, and competitive populations.

### 4.5. Practical Applications and Perspectives

The present findings may have practical relevance for sprint finswimming training. The faster performance achieved during UW, together with fewer dolphin kicks and a comparable kick frequency, suggests that coaches should consider not only kicking rate but also the overall organization of underwater movement when designing sprint-specific training. Underwater and apnea-based sprint drills may therefore be useful for practicing the specific technical and physiological demands of high-intensity submerged swimming.

From a physiological perspective, the biphasic HR response observed during early recovery may provide useful descriptive information when monitoring athletes following maximal apnea efforts. Tracking the early post-exercise HR pattern may complement conventional recovery monitoring, particularly in athletes who regularly perform breath-hold or underwater sprint training. However, the practical significance of this response remains to be established. Future intervention studies should determine whether repeated underwater or dynamic-apnea training can improve sprint performance, influence recovery characteristics, or modify physiological tolerance over time.

## 5. Conclusions

The present study showed that 50 m sprint performance was significantly faster during underwater apnea swimming than during the two surface swimming conditions in highly trained young male national-level finswimmers. UW was also characterized by a lower number of dolphin kicks, whereas kick frequency remained comparable across conditions. These findings are consistent with potential biomechanical and hydrodynamic explanations described in previous research. However, hydrodynamic resistance, propulsive efficiency, and other detailed biomechanical variables were not directly measured, and the mechanisms responsible for the superior UW performance therefore cannot be established from the present data. A biphasic HR recovery pattern was observed across all three conditions, characterized by an initial post-exercise decline followed by a secondary increase during early recovery. This should be interpreted as an observed temporal HR response rather than as evidence of a specific autonomic mechanism. No statistically significant between-condition differences were detected in peak HR, blood lactate concentration, blood glucose concentration, or perceived exertion. Further studies incorporating direct biomechanical, hydrodynamic, autonomic, respiratory, and oxygenation measurements are required to determine the mechanisms underlying the observed performance and HR responses.

## Data Availability

The data presented in this study are openly available in FigShare at https://doi.org/10.6084/m9.figshare.33094337.

## Abbreviations

The following abbreviations are used in this manuscript:

BG	Blood glucose concentration
BL	Blood lactate concentration
CO_2_	Carbon dioxide
FS	Finswimming
HIF-1α	Hypoxia-inducible factor-1α
HR	Heart rate
HRhigh	Subsequent higher heart rate during early recovery
HRlow	Minimum heart rate during early recovery
HRpeak	Peak heart rate during the swimming trial
DKF	Dolphin kick frequency
DKN	Dolphin kick number
MF	Monofin swimming
RPE	Rating of perceived exertion
SC	Surface swimming using a closed snorkel
SN	Surface swimming without a snorkel
SP	Swimming performance
UW	Underwater swimming
